# Dosimetric Analysis of Fast-Forward Breast Radiotherapy Using 3D-Conformal Radiotherapy (3D-CRT) With Deep Inspiratory Breath Hold (DIBH)

**DOI:** 10.7759/cureus.93746

**Published:** 2025-10-02

**Authors:** Ajithkumar M, Subhankar Show, Aaditya Prakash

**Affiliations:** 1 Radiation Oncology, Meherbai Tata Memorial Hospital, Jamshedpur, IND; 2 Radiotherapy, Meherbai Tata Memorial Hospital, Jamshedpur, IND; 3 Radiation Oncology, Tata Main Hospital, Jamshedpur, IND

**Keywords:** 3d-conformal radiotherapy (3d-crt), cardiac sparing, deep inspiration breath hold (dibh), dosimetric analysis, hypofractionated radiotherapy, left-sided breast cancer, voluntary breath hold

## Abstract

Introduction and aim

Fast-forward radiotherapy (26 Gy in five fractions) has gained widespread acceptance for early-stage breast cancer due to its convenience and efficacy. However, in left-sided cases, there is a risk of radiation-induced cardiac and pulmonary toxicity due to the proximity of the heart and lungs. The voluntary deep inspiration breath-hold (vDIBH) technique increases thoracic volume and heart-chest wall distance, offering a non-invasive method to minimize organ-at-risk exposure. This study aimed to evaluate the dosimetric benefits of 3D-conformal radiotherapy (3D-CRT) combined with voluntary deep inspiration breath hold (vDIBH) in minimizing radiation dose to the heart and lungs during ultra-hypofractionated whole-breast irradiation in patients with left-sided breast cancer.

Materials and methods

We retrospectively analyzed 28 patients with left-sided breast cancer treated with 3D-CRT under vDIBH, receiving 26 Gy in five fractions. Target volumes and organs at risk (OARs) were contoured on vDIBH CT scans. Treatment planning was performed using tangential fields. Dosimetric parameters for the planning target volume (PTV), heart, ipsilateral lung, contralateral breast, and spinal cord were evaluated using dose-volume histograms (DVHs). Reproducibility of vDIBH was ensured by using in-room lasers, skin tattoos, and cine-mode imaging for intrafraction verification.

Results

Mean PTV V95% was 97.74±1.74%, with a mean dose of 26.61±0.25 Gy. The mean heart dose was 3.97±0.82 Gy, with V25% at 13.28±3.44%. Ipsilateral lung mean dose (D_mean_) was 8.26±0.76 Gy, and V30% was 32.81±2.85%. Contralateral breast V5% was 5.19±7.06%. The spinal cord received a negligible dose (maximum dose {D_max_}​​​​ 0.49±0.12 Gy). All patients tolerated vDIBH well, with no delays or treatment interruptions.

Conclusion

3D-CRT with vDIBH offers a practical, reproducible, and cost-effective approach for delivering hypofractionated radiotherapy in left-sided breast cancer. It ensures excellent target coverage while significantly reducing radiation dose to critical structures, such as the heart and lungs, supporting its use as a standard practice even in resource-constrained settings.

## Introduction

Postoperative radiation therapy (RT) is a cornerstone in the management of early-stage breast cancer, significantly reducing the risk of local recurrence and improving overall survival [1-8]. In recent years, hypofractionated regimens, such as fast-forward breast radiotherapy (26 Gy in five fractions over one week), have become increasingly popular due to their convenience, reduced treatment time, and comparable efficacy to conventional schedules. However, in patients with left-sided breast cancer, the close anatomical proximity of the heart and left anterior descending (LAD) artery to the chest wall presents a substantial risk of radiation-induced cardiac toxicity. Several large studies have demonstrated that cardiac doses from breast cancer radiotherapy are generally higher for left-sided than for right-sided tumors, with long-term data indicating that the cumulative risk of cardiac death at 20 years is 6.4% for left-sided cases compared to 3.6% for right-sided counterparts [8-24].

To mitigate this risk, various strategies have been developed, among which the voluntary deep inspiration breath-hold (vDIBH) technique has emerged as a simple, reproducible, and effective method. vDIBH involves asking the patient to take and hold a deep breath during radiation delivery, which expands the lungs and moves the heart inferiorly and posteriorly, thereby increasing the distance between the heart and the treatment field [2,3,7]. This maneuver has been shown to substantially reduce the volume of the heart and LAD artery receiving significant radiation doses. McIntosh et al. reported that use of vDIBH in 3D-conformal whole-breast radiotherapy resulted in a 7% reduction in mean heart dose and a 9% reduction in mean LAD dose compared to free breathing [20].

In addition to reducing cardiac dose, vDIBH has also been associated with a decrease in the volume of lung irradiated, thereby minimizing the risk of pulmonary toxicity [2,3]. The vDIBH technique, in particular, has gained traction in clinical settings because it is simple to implement, requires no specialized external equipment, and is cost-effective. With appropriate patient coaching and immobilization, this technique can achieve reproducible breath-hold levels and stable anatomy throughout treatment [3-5].

Despite advances in highly conformal techniques like intensity-modulated radiation therapy (IMRT), issues such as sensitivity to intrafraction motion, coverage of the flash region, and increased low-dose exposure remain concerns, particularly in breast treatment [6,9,10]. In contrast, 3D-conformal radiotherapy (3D-CRT) combined with vDIBH provides a balanced approach by maintaining adequate target coverage while effectively sparing organs at risk. Furthermore, the field-in-field (FIF) technique, a variation of 3D-CRT that uses additional subfields with manually shaped apertures, improves dose homogeneity, reduces acute skin toxicity, and enhances cosmetic outcomes [6,9,11-15].

Cardiac toxicity from breast RT is both technique-dependent and dose-volume-dependent, and the use of cardiotoxic systemic therapies (e.g., anthracyclines, trastuzumab, taxanes) further underscores the importance of minimizing radiation dose to the heart [10,14]. The vDIBH technique allows for substantial sparing of the heart without compromising target coverage or increasing dose to the contralateral breast [11]. As such, it has been adopted as the standard of care for left-sided breast cancer RT in many institutions.

This study, titled "dosimetric analysis of fast-forward breast radiotherapy using 3D-CRT with deep inspiration breath hold (DIBH)," presents a retrospective dosimetric analysis of patients treated with 3D-CRT using the vDIBH technique, aimed at reducing radiation dose to organs at risk.

## Materials and methods

Patient selection

In this study, 28 patients with left-sided breast cancer who underwent mastectomy followed by irradiation to the whole breast using an ultra-hypofractionated regimen (26 Gy in five fractions over one week) were reviewed retrospectively. All patients had early-stage disease, classified as T1-T2, N0, M0 based on the American Joint Committee on Cancer Tumor, Node, Metastasis (AJCC TNM) staging system [19]. Patient ages ranged from 35 to 60 years, with a median age of 47 years. Regional lymph nodes, including the supraclavicular, undissected axillary, and internal mammary (IM) nodes, were not included in the treatment volumes. All relevant target volumes were contoured on vDIBH planning CT scans, and treatment planning was performed using the Eclipse Treatment Planning System version 15.6 (Palo Alto, CA: Varian Medical Systems), with a 3D-CRT technique with tangential fields used to cover the breast tissue [7].

Patient positioning and simulation

All patients were positioned on an inclined breast board with both arms raised above the head and immobilized using dedicated arm supports to ensure reproducibility during simulation and treatment [1]. The borders of the tangential treatment fields were determined clinically by the radiation oncologist and marked with radiopaque wires. CT simulation scans were acquired using a 3 mm slice thickness, covering the region from the mid-neck to the mid-abdomen [14].

Laser alignment and breath-hold reproducibility

To verify and monitor chest wall excursion during vDIBH, an additional laser was mounted on a tripod to avoid interference from the gantry. A bright ink marker line was drawn on the patient’s skin posterior to the tattooed alignment mark, based on the depth measured during CT simulation. During deep inspiration, proper alignment of the laser with the ink mark confirmed consistent breath-hold positioning. This alignment was observed remotely via a magnification camera by the radiation therapist throughout beam delivery. If misalignment (e.g., indicating expiration) was observed, the beam was manually interrupted to maintain accuracy [2].

Patient compliance and training

Prior to CT simulation, all patients were educated about the vDIBH technique, as it requires active patient participation to achieve and maintain consistent breath holds during each treatment field. Patients were instructed to breathe in and out twice, then take a deep breath and hold it for 20 seconds during the simulation. A vDIBH planning CT and a corresponding free-breathing (FB) CT were acquired for each patient for comparison and planning purposes [3,11].

Treatment planning and setup

The initial treatment setup was based on skin markings and alignment with in-room lasers. The difference in inspiration depth between FB and vDIBH was measured and marked on the patient's skin. During each treatment, the patient was instructed via audio-visual communication from the treatment console to breathe in and hold until the skin mark aligned with the in-room laser. The radiation therapists observed breath-hold positioning via a live video feed and instructed the patient accordingly. In addition to routine, an electronic portal imaging device (EPID) was operated in cine mode to capture a sequence of images during beam-on time for intrafraction verification [3]. All patients received the prescribed dose of 26 Gy in five fractions using 3D-CRT with two tangential fields. Plans were created using a combination of 6 MV and 10 MV photon beams to optimize target coverage while minimizing dose to the heart and ipsilateral lung. Dose constraints were analyzed to quantify the benefit of vDIBH in sparing cardiac and pulmonary structures [13].

CT image matching and planning target volume (PTV) margin calculation

We confirm that the margin calculation was performed retrospectively, based on setup error analysis from cone-beam computed tomography (CBCT) compared with planning CT. The planning target volume (PTV) margins were calculated using the van Herk formula as follows: margin=2.5Σ+0.7δ, where Σ represents the systematic error and δ represents the random error [6,9,12,13,16,17].

Dosimetric evaluation

The dosimetric parameters evaluated in this study included V95%, V105%, V107%, and D_max_ for PTV, which reflect target coverage. For the heart, key parameters analyzed were V5%, V25%, and mean dose, providing insights into low- and high-dose cardiac exposure. The left lung was assessed using V30% and mean dose to evaluate pulmonary dose burden. Additionally, V5% was measured for the right breast to quantify incidental exposure and assess the risk of contralateral dose spillage.

## Results

Dosimetric outcomes were evaluated for 28 patients with left-sided breast cancer treated with 3D-CRT under vDIBH, prescribed to 26 Gy in five fractions. Dose volume histogram data were extracted from the treatment planning system. The detailed dosimetric parameters for the PTV and organs at risk are summarized in Table 1.

**Table 1:** Dosimetric parameters for target and organs at risk in patients treated with 3D-CRT using vDIBH. V5%, V25%, and V30% percentage volume receiving at least 5%, 25%, or 30% of the prescribed dose, respectively. All dose values are expressed in Gy. Values are presented as mean±standard deviation. PTV: planning target volume; vDIBH: voluntary deep inspiration breath hold; 3D-CRT: 3D-conformal radiotherapy; V95%: volume of PTV receiving ≥95% of the prescribed dose; V107%: volume of PTV receiving ≥107% of the prescribed dose; D_mean_: mean dose; D_max_: maximum dose

Structures	Metrics	Results
PTV	V95%	97.74±1.74
PTV	V107%	6.31±5.76
PTV	D_mean _(cGy)	26.61±0.25
Heart	V5%	40.72±12.98
Heart	V25%	13.28±3.44
Heart	D_mean _(cGy)	3.97±0.82
LT lung	V30%	32.81±2.85
LT lung	D_mean _(cGy)	8.26±0.76
RT breast	V5%	5.19±7.06
Spinalcord	D_max _(cGy)	0.49±0.12

Target volume coverage

The PTV demonstrated robust coverage, with V95% reaching 97.74±1.74%, thereby meeting the Radiation Therapy Oncology Group (RTOG) 1005 protocol criteria. The mean dose to the PTV was 26.61±0.253 Gy, which aligns precisely with the prescribed dose. Additionally, V107% was 6.31±5.76%, reflecting an acceptable degree of dose inhomogeneity consistent with field-in-field modulation techniques.

Heart dose parameters

The application of vDIBH resulted in effective cardiac sparing. The volume of the heart receiving at least 5% of the prescribed dose (V5%) was 40.72±12.98%, while V25% was limited to 13.28±3.44%. The mean heart dose was 3.98±0.83 Gy, which remains well below the 5.0 Gy constraint recommended for ultra-hypofractionated breast radiotherapy, as outlined by RTOG 1005 [18].

Left lung dose parameters

Due to the use of tangential field geometry, moderate involvement of the ipsilateral lung was observed. The V30% of the left lung was 32.81±2.85%, and the mean dose was 8.27±0.77 Gy. These values are consistent with expectations for conformal planning in breast radiotherapy.

Contralateral breast dose

Scatter radiation to the contralateral breast was minimal, with a V5% of 5.19±7.06%. This indicates effective sparing of the opposite breast and helps reduce the long-term risk of radiation-induced secondary malignancies.

Spinal cord dose

Although the spinal cord was not a direct area of interest in this study, it was included for completeness. The maximum dose (D_max_) to the spinal cord was negligible, measured at just 0.49±0.13 Gy, which is well within established safety thresholds.

**Figure 1:**
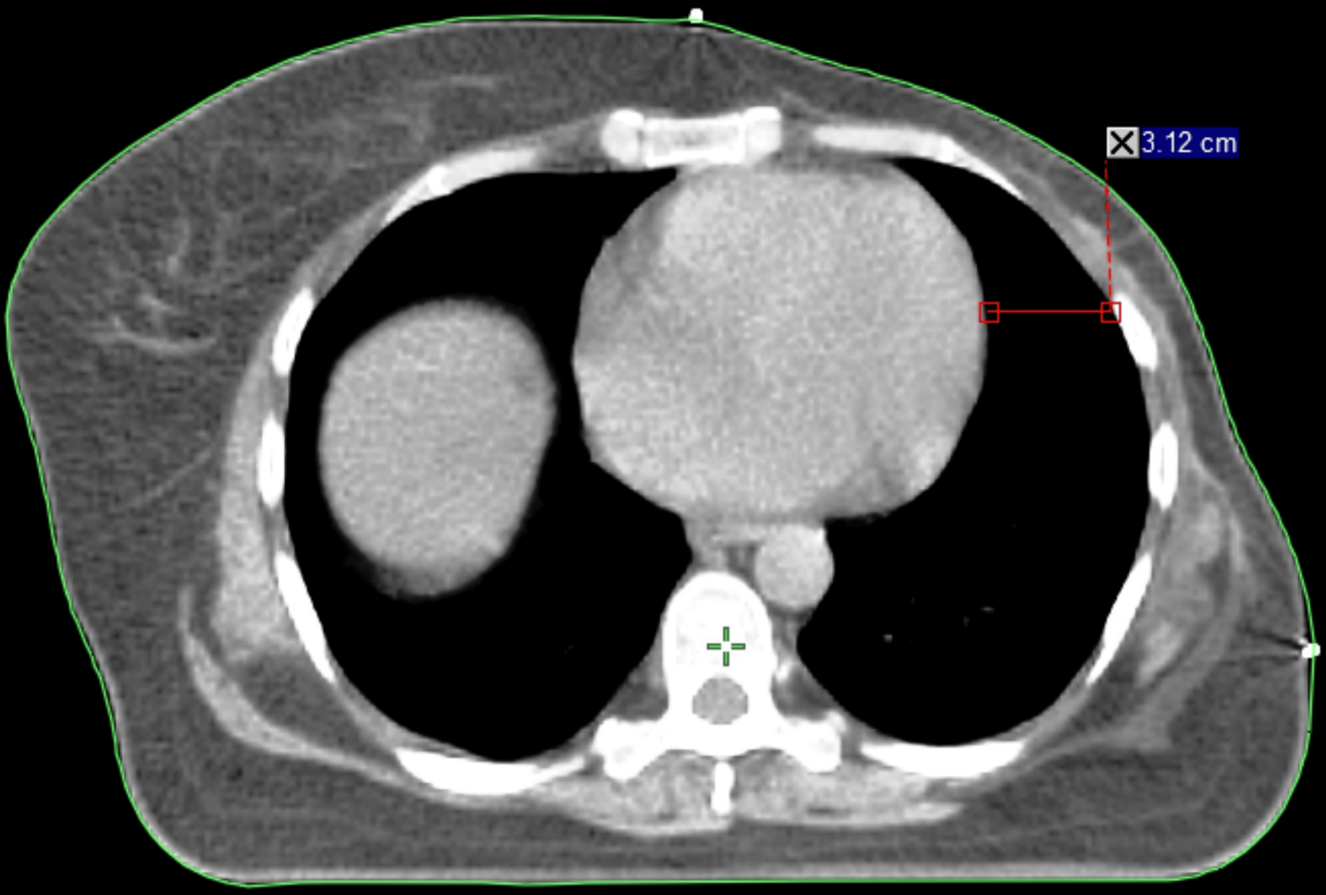
CT image acquired during FB. This axial CT shows that the heart-chest wall distance is reduced to 3.12 cm. The heart lies closer to the left chest wall and within the high-dose region, underscoring the need for heart-sparing techniques in left-sided breast radiotherapy. FB: free breathing

**Figure 2:**
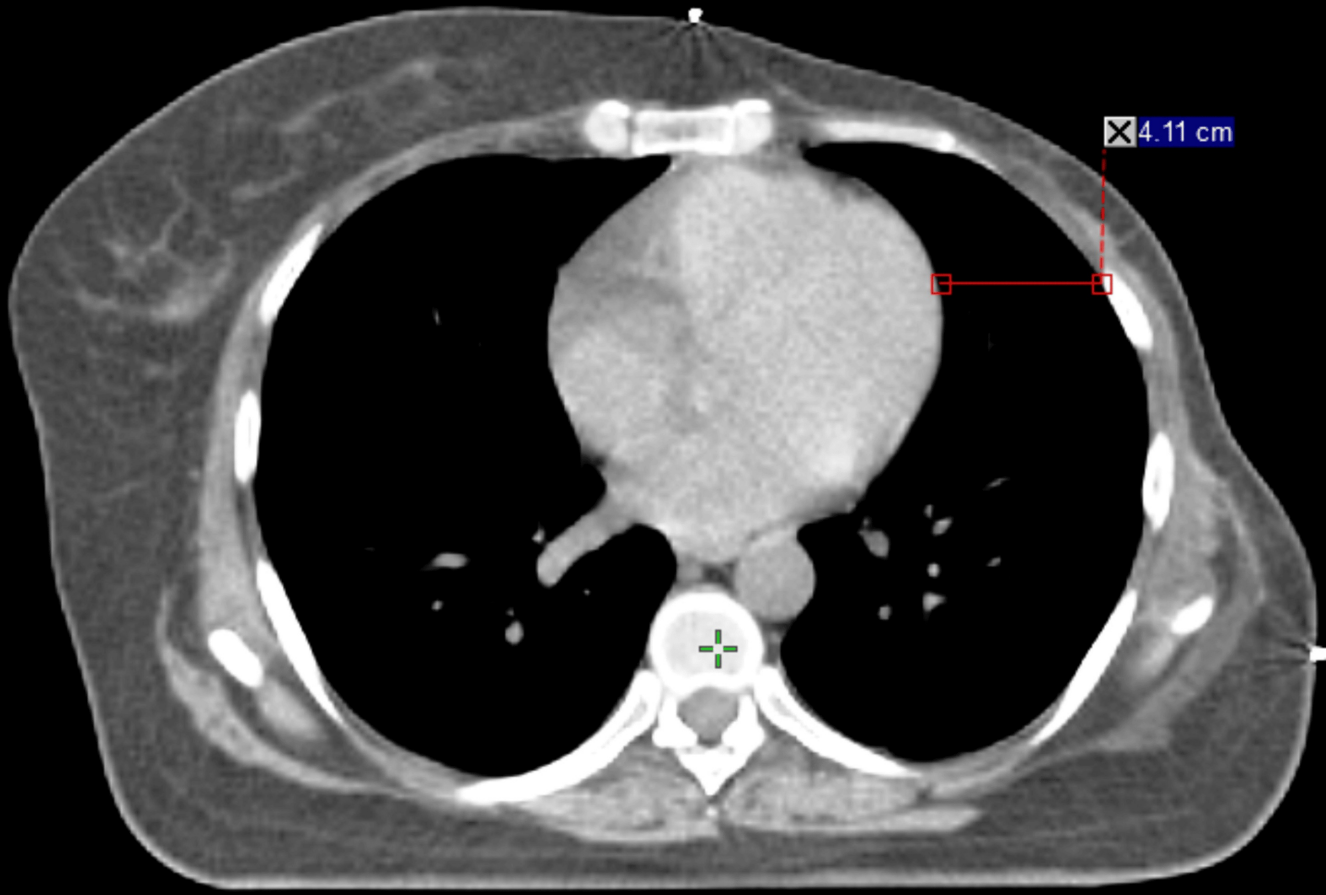
CT image acquired during vDIBH. This axial planning CT shows an increased separation of 4.11 cm between the anterior surface of the heart and the chest wall, demonstrating the effectiveness of vDIBH in displacing the heart posteriorly and inferiorly, away from the tangential radiation fields. vDIBH: voluntary deep inspiration breath hold

**Figure 3:**
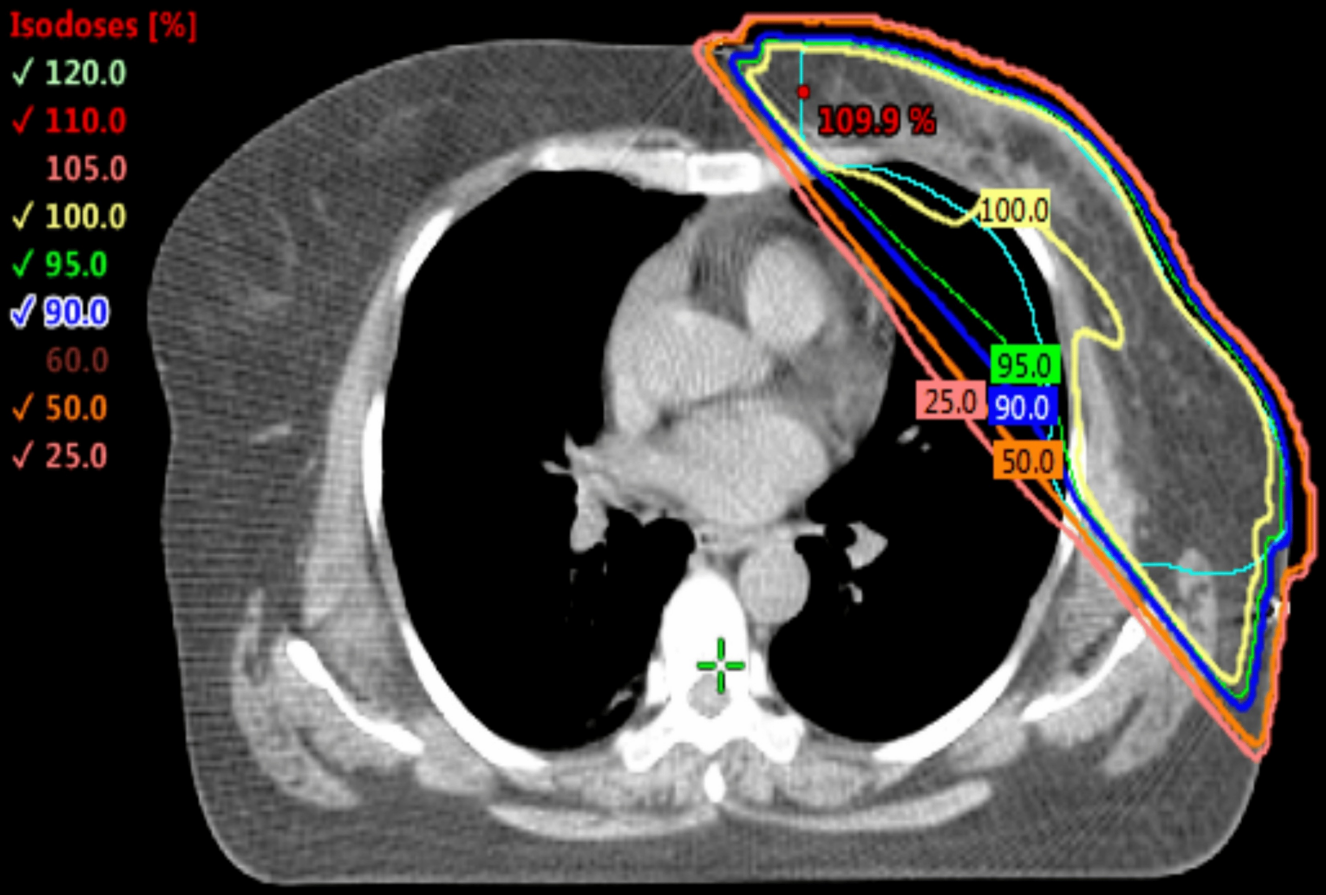
Isodose distribution from a 3D-CRT plan using vDIBH. The axial isodose map demonstrates homogeneous dose coverage to the planning target volume (PTV) with effective sparing of the heart and ipsilateral lung, confirming the benefit of combining 3D-CRT with vDIBH for cardiac dose reduction. vDIBH: voluntary deep inspiration breath hold; 3D-CRT: 3D-conformal radiotherapy

The dosimetric benefits of voluntary deep inspiration breath hold (vDIBH) are clearly illustrated in Figures 1, 2, which demonstrate how thoracic expansion during vDIBH increases the separation between the heart and anterior chest wall, leading to reduced radiation exposure to both the heart and ipsilateral lung. Figure 3 presents the corresponding isodose distribution, where the dose lines reflect excellent target coverage while substantially sparing critical organs. Collectively, these figures highlight how vDIBH enables favorable isodose shaping around the treatment target without compromising organ-at-risk protection.

## Discussion

This study demonstrates that 3D-CRT combined with vDIBH is an effective and practical technique for delivering ultra-hypofractionated whole-breast radiotherapy in patients with left-sided breast cancer. The use of vDIBH allowed for robust target coverage while significantly reducing radiation exposure to the heart and ipsilateral lung. Our findings align with previous studies showing that vDIBH increases the distance between the heart and chest wall, thereby lowering cardiac doses. Compared to the pooled average heart dose of 4.7 Gy reported in earlier DIBH studies, our patients achieved slightly lower mean values, further confirming the dosimetric benefit of this approach [12]. Lung sparing was also achieved without compromising target coverage, in agreement with prior literature demonstrating reduced lung dose through thoracic expansion during deep inspiration.

Long-term data from the Early Breast Cancer Trialists’ Collaborative Group (EBCTCG) showed that patients treated with radiotherapy in the 1960s and 1970s experienced a 30% increase in cardiac mortality [2,6,9]. Although modern CT-based planning techniques have reduced unnecessary cardiac exposure, studies with over 10 years of follow-up are still needed to assess the true incidence of late cardiac events [1]. Darby et al. reported that each 1 Gy increase in mean heart dose is associated with a 7.4% increase in the rate of major coronary events, including myocardial infarction and cardiac death [21]. Similarly, Sardaro et al. estimated a 4% increase in the risk of late cardiac disease per 1 Gy [22]. Our approach to vDIBH implementation involved a simple, low-cost setup using in-room lasers, skin tattoos, and cine-mode imaging for intrafraction verification. This ensured reproducible breath-hold positioning without requiring expensive respiratory monitoring equipment. Patient tolerance and compliance were excellent, and breath-hold reproducibility was maintained throughout treatment. No increase in treatment time or workflow disruption was observed, supporting the feasibility of incorporating vDIBH into standard radiotherapy practices even in resource-limited centers.

While the heart is the primary concern in left-sided breast radiotherapy, minimizing dose to the ipsilateral lung is also critical [10]. Our data confirmed that vDIBH also achieved effective lung sparing, consistent with prior reports. Additionally, the contralateral breast dose remained low. Although the long-term impact is still debated, some evidence, such as from atomic bomb survivor data, suggests that doses between 1 and 4 Gy may modestly increase the risk of contralateral breast cancer after more than 15 years [23]. Visual coaching and feedback methods, such as video goggles, have been shown to further enhance breath-hold reproducibility and treatment accuracy in left-breast cancer patients undergoing DIBH [16]. These techniques may offer additional benefits when integrated into standard workflows.

However, this study is limited by its retrospective design, relatively small sample size, and absence of a direct free-breathing comparison. Clinical outcomes, such as cardiac toxicity, secondary malignancies, or cosmetic effects, were not evaluated. Only routinely contoured organs at risk (heart and ipsilateral lung) were analyzed; detailed contouring of the left ventricle (LV) and left anterior descending (LAD) artery was not performed, which is a limitation. Future prospective studies with larger cohorts and long-term follow-up are warranted to validate these dosimetric benefits and assess their clinical significance. In summary, the findings of this study support the clinical utility of 3D-CRT with vDIBH, which offers a simple, effective, and resource-efficient method for delivering hypofractionated left-sided breast radiotherapy while minimizing radiation dose to critical organs. Its ease of implementation, patient compliance, and cost-effectiveness support its broader adoption in modern clinical practice.

## Conclusions

The combination of 3D-CRT with vDIBH has demonstrated clear dosimetric and clinical advantages in the treatment of left-sided breast cancer. This technique offers excellent target coverage while significantly reducing radiation exposure to the heart and lungs, which is critical for minimizing long-term cardiopulmonary risks. Our approach utilized a cost-effective and easily implementable setup involving in-room lasers, skin tattoos, and cine-mode imaging for intrafraction verification. This ensured reproducible breath-hold positioning without the need for expensive respiratory gating systems or prolonging treatment times. The technique was well tolerated by patients and did not impact daily throughput, underscoring its feasibility in busy clinical environments. The safety, simplicity, and effectiveness of vDIBH support its routine use in left-sided breast radiotherapy, including in resource-limited settings. Broader adoption of this approach can improve treatment quality, reduce toxicity, and contribute to better long-term outcomes for breast cancer patients. However, despite the dosimetric benefits, the risk of cardiac toxicity from residual heart exposure remains an important consideration. This underscores the need for careful treatment planning and continued efforts to optimize heart-sparing while maintaining target coverage.
